# Clinical Notes—Dysmenorrhœa

**Published:** 1887-01

**Authors:** J. T. Kent

**Affiliations:** St. Louis


### CLINICAL NOTES.—DYSMENORRHŒA.

PROFESSOR J. T. KENT, M. D., ST. LOUIS.

Mattie E——, æt. twenty-three. Since the first menstrual nisus, which occurred at thirteen, she has suffered great pain at every period, which has been every three weeks. Pain in the uterus and down the limbs. Before and during she has suffered from an empty, hungry, all-gone feeling in the stomach (Sepia, Murex, Ignatia); she cannot stand long on the feet, the pain is so much aggravated; cold feet, great dizziness when going up stairs, voracious appetite.

The fact that this difficulty dated back to puberty guided me to Calc. phos. She never had any more pain. This young lady was compelled to avoid any engagement that might come on her sick day, as she was compelled to keep her bed most of the first day. Her expressions of gratitude have often cheered me, and her praise has brought me much business.

So important is Calcaria phos. in the painful affections of the uterus connected with puberty, and resulting from bad habits or neglected advice at that time, that I feel like emphasizing this feature of it. It is a common practice in rural districts for girls at puberty to wade in water and do many careless things, thereby laying foundation for dysmenorrhœa and sterility. The complaints growing out of these causes find their remedy in Calc. phos. in a very large number of instances.

Miss X——, twenty-four years old, had suffered from *dysmennorrhœa* since puberty. She always kept her bed during the first day. Menses a few days too soon and profuse, lasting five days. The pain was labor-like, and there was some bearing down in the vagina, with a sensation as if the parts would protrude. She often felt as if her menses would come on at different times during the interim, and sometimes a sexual flame annoyed her. Generally she was robust and free from complaint. Calc. phos. cured this lady in two months.

She was an orphan, having no mother to advise her, therefore exposure at the time that she most needed to exercise judgment, brought on the suffering that lasted ten years before she obtained the appropriate remedy. This patient had submitted to local treatment without palliation. She had been told that internal medication could not benefit her.

Miss Susie C——, twenty-two years old, consulted me for dysmenorrhœa. Her menses came very much too soon, and lasted from seven to ten days. The flow was dark and clotted the first three or four days; the severe pain was at the beginning; she got some relief after passing membranes. She complained of aphthous patches in the mouth and sometimes on the labia. She always had a leucorrhœa several days before menstruation, white-of-egg-like and ropy. Her pains were often labor-like, constricting (Cactus), extending into the back and up the back (Gels.), and down the thighs (Cham.), and sometimes to the stomach, causing vomiting. She would always weep from music (Natrum) and grow sick and become frightened when going down from the top of any high building in an elevator.

She got Borax$^{3m}$ at proper intervals. The result was satisfactory. The second period was painless and normal. The relief in this case has been permanent.

